# Epidemiology, pathogenicity, risk factors, and management of *Helicobacter pylori* infection in Saudi Arabia

**DOI:** 10.17305/bb.2023.9575

**Published:** 2024-06-01

**Authors:** Mutasim E Ibrahim

**Affiliations:** 1 Department of Basic Medical Sciences, College of Medicine, University of Bisha, Bisha, Saudi Arabia

**Keywords:** *Helicobacter pylori*, prevalence, pathogenicity, gastric cancer, risk factors, management, Saudi Arabia

## Abstract

*Helicobacter pylori* (*H. pylori*) is a gastric microbial pathogen that infects approximately half of the global population. This bacterium significantly contributes to various gastroduodenal diseases, including chronic active gastritis, peptic ulcerations, and malignant transformations. This review focuses on the epidemiology, pathogenicity, virulence genes, risk factors, and management of *H. pyl*ori infection, specifically within the context of Saudi Arabia. The results presented here are grounded in studies conducted in Saudi Arabia, contrasting with mere bibliographic reviews of findings from other countries. *H. pylori* infection has been observed in Saudi Arabia, with substantial differences in the prevalence, ranging between 10% and 96% among various studied populations. Several risk factors for *H. pylori* infection have been identified, encompassing socioeconomic status, medical history, personal hygiene, and behavioral practices. Among the virulence genes harbored by *H. pylori*, cytotoxin-associated gene A (*cagA*), and vacuolating cytotoxin (*vacA*) are the most common, with their presence correlating with the pathogenicity and clinical manifestations of the associated diseases. A range of invasive and non-invasive diagnostic assays have been utilized to identify *H. pylori* infection, with their employment being influenced by factors like availability, cost, patient age, gastric symptoms, and the specifics of clinical information sought. While detection methods like the *H. pylori* stool antigen test and the urea breath test offer more accuracy and speed, culturing remains indispensable for determining the antimicrobial susceptibility profile. The emergence of resistant strains across varying regional settings poses a significant challenge to treatment endeavors, necessitating an assessment of local antimicrobial resistance rates prior to formulating treatment strategies. The findings of this review highlight the importance of continuous implementation of screening, control, and prevention of *H. pylori* infection to combat the spreading infection and other related complications.

## Introduction

*Helicobacter pylori* (*H. pylori*) is a spiral, non-sporing Gram-negative, unipolar, multiflagellate microaerophilic bacterium, recognized in animal stomachs as early as 1893 [1, 2]. In 1983, Marshall and Warren isolated it as a major etiological agent of stomach inflammation [3]. Although *H. pylori* was only identified in 1983, it has become the most prevalent gastric microbial pathogen, with half or more of the world’s population becoming infected [2, 4, 5]. Acquisition of *H. pylori* primarily happens during childhood, and once acquired, the infection persists throughout life unless specifically treated [3]. *H. pylori* colonizes the gastric mucosa of humans and plays a significant role in the pathogenesis and development of peptic ulcers [6, 7]. Infection with *H. pylori* stimulates various upper gastrointestinal tract (GIT) diseases, ranging from dyspeptic symptoms, chronic gastritis, and peptic ulcer to gastric cancer [2, 5, 7–11]. The annual cost associated with peptic ulcer diseases in the United States is estimated to be $6 billion, and gastric cancer kills over 700,000 people per year globally [12]. Research evidence indicates that *H. pylori* is responsible for 74% of non-cardia gastric cancer in developed countries and 78% in less developed countries [13]. In developing countries, over 3 billion people, accounting for roughly 50% of the world’s population have been affected by *H. pylori* infection in their life, 17% develop peptic ulcer that progresses to gastric cancer in 1% [14].

Due to its widespread prevalence, *H. pylori* remains a challenging worldwide medical problem [15]. The prevalence varies worldwide and depends on the economy of each country, the patient’s gender, ethnic background, and the socioeconomic conditions of the population [5, 16]. In developed countries, the prevalence of *H. pylori* varies between 25% and 50%, while in developing countries, it increases to 90% [8, 16, 17]. In Middle Eastern countries, the prevalence among the adult population is in the range of 70%–90% [17]. Therefore, understanding the epidemiology of *H. pylori* infection concerning the geographical distribution and sociodemographic characteristics is necessary to develop effective public health measures and prevent the spread of infection caused by this bacterium [6]. This article reviews the epidemiology, pathogenicity, risk factors, virulence genes, antimicrobial susceptibility, and diagnostic methods of *H. pylori* infection in Saudi Arabia.

## Materials and methods

**Figure 1.**
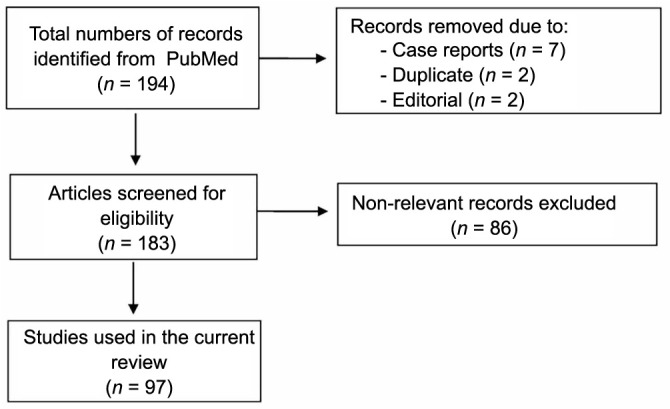
Flowchart of the literature search and selection process of the studies.

A literature search was conducted in the PubMed database to obtain potential articles on *H. pylori* infection in Saudi Arabia between January 1990 and December 2022. The search terms with Medical Subject Headings (MeSH) used to collect relevant studies were “*Helicobacter pylori*” OR “*H. Pylori*” OR “*Helicobacter* infections/epidemiology” OR “*Helicobacter* infections/pathology” OR “*Helicobacter* infections/genetics” OR “*Helicobacter* infections/diagnosis” OR “*Helicobacter* infections/drug therapy” AND “Saudi Arabia.” The articles were screened for eligibility based on the title, abstract, and keywords. Bibliographies of eligible studies were manually searched to identify additional articles and to avoid missing any relevant articles. The search was limited to articles written in English with abstract or full texts. Case reports, editorial materials, and conference papers were excluded. Search results were imported into Mendeley Desktop, and duplicate citations were removed manually. The procedure above resulted in 97 articles (Figure 1).

## Results

### Prevalence of *H. pylori* infection in Saudi Arabia

**Figure 2.**
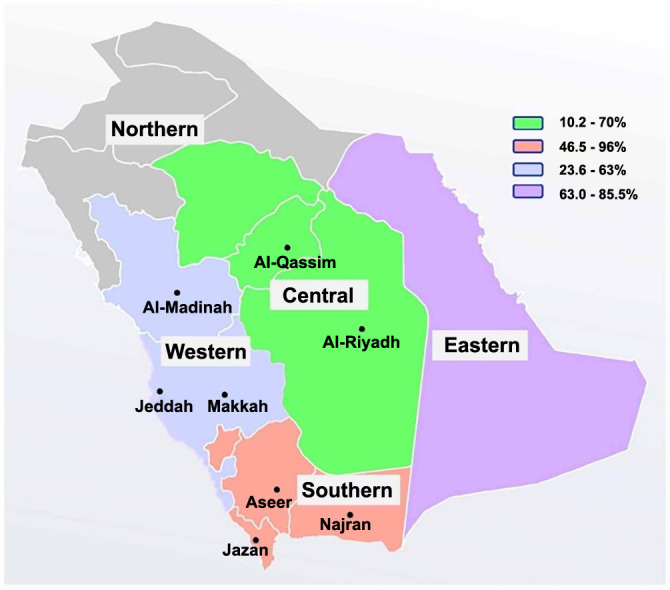
Map of Saudi Arabia showing the prevalence rates of *H. pylori* infection in different geographical regions.

Administratively, Saudi Arabia is divided into 13 regions, which are distributed in the five geographical areas of the country (Figure 2). Most of the prevalence studies of *H. pylori* infection were conducted in the central, southern, and western regions, with a few reports from the eastern area (Table 1). The infection of *H. pylori* has been observed earlier in Saudi Arabia, with substantial differences in the prevalence of the infection between geographical areas and with the studied population. The disease prevalence range between 10.2% and 96% has been estimated in the different geographical locations of the country using various laboratory assays and sample sizes [7, 18]. Most of the published data in the country determined the prevalence of *H. pylori* among asymptomatic or symptomatic patients in relation to their sex, age, and associated comorbidities. Table 1 presents data from the prevalence studies published in Saudi Arabia since 1991. High rates of *H. pylori* infection have been reported in earlier studies in different geographical regions of Saudi Arabia, but these rates have decreased over time.

**Table 1:** Prevalence of *H. pylori* infection determined in studies from 1990 to 2022 in different geographical regions in Saudi Arabia

**Area**	**Study sample**	**Study subjects**	**Sample size**	**Year of sampling**	**Sample type**	**Age range (years)**	**Test method**	**Prevalence (%)**	**References**
*Central*									
Riyadh	Community-based	General individuals	557	1990	Blood	5–91	Serology	40–70	[28]
Riyadh	Histopathology-based	Patients with dyspepsia	352	1991	Gastric biopsy	17–69	Histology	61.64	[29]
Riyadh	Urban-based	Outpatient with GI symptoms	5782	2007	Blood	2–82	Serology	67	[30]
Riyadh	Histopathology-based	Children with GI symptoms	303	2010–2013	Gastric biopsy	<14	Histology	49.8	[65]
Riyadh	Community-based	Healthy school children	3551	2014–2016	Blood	6–15	Serology	40	[31]
Riyadh	Urban-based	Patients with GI symptoms	411	2018	Stool	14–64	Stool antigen	10.2	[7]
Riyadh	Histopathology-based	Patients undergoing bariatric surgery	356	2014–2016	Gastric biopsy	15–66	Routine lab. tests	41	[33]
Riyadh	Histopathology-based	Patients with dyspepsia	1398	2012–2016	Biopsy	11–95	Histology	34.7	[34]
Riyadh	Community-based	Medical students	120	2005	Breath	18–28	Urea breath	35	[8]
Qassim	Community-based	Student population	554	2007–2008	Blood	16–18	Serology	51	[32]
Qassim	Urban-based	Patients with dyspepsia and epigastric pain	810	2020–2021	Stool	37.68 ± 18.7 (Mean ± SD)	Stool antigen	24.9	[35]
*West*									
Madinah	Community-based	Student population	436	2007–2008	Blood	16–18	Serology	42	[32]
Madinah	Histopathology-based	Patients with gastric diseases	1236	2006–2015	Gastric biopsy	10–100	Histology	32.5	[104]
Madinah	Urban-based	Patients with renal failure	127	2021	Blood	≥10	Serology	33.1	[105]
Makkah	Community-based	Healthy population	396	2003	Blood	15–50	Serology	51	[16]
Makkah	Histopathology-based	Patients with peptic ulcer disease	132	2003–2004	Gastric biopsy	14–90	Urease test Histology Culture Serology	63	[6]
Makkah	Community-based	School students	314	2008	Breath	12–18	Urea breath	27.4	[37]
Makkah	Urban-based	Patients with epigastric discomfort	100	2015	Blood	47.17 ± 9.2 (Mean ± SD)	Serology	62	[4]
Makkah	Histopathology-based	Patients with suggestive chronic gastritis	368	2004–2005	Gastric biopsy	16–90	Culture PCR	28	[38]
Makkah	Urban-based	Patients with iron deficiency anemia	79	2018–2020	Blood	21–68	Serology	62	[39]
Jeddah	Community-based	Boys of intermediate school	132	Not mentioned	Breath	12–15	Urea breath	51.5	[43]
Jeddah	Histopathology-based	Symptomatic children	303	2010–2013	Stool Blood Gastric biopsy	Children of all age groups	Urease test Antral nodularity Histology Biopsy culture Serology Stool antigen	49.8	[42]
Jeddah	Urban-based	Children	1432	2001–2003	Blood	≥1.0	Serology	23.6	[9]
Taif	Histopathology-based	Patients with dyspepsia	680	2019–2021	Gastric biopsy	12–97	Histology	32.5	[41]
*South*									
Aseer	Community-based	Student population	467	2007–2008	Blood	16–18	Serology	50	[32]
Aseer	Histopathology-based	Patients with GI symptoms	208	1992	Gastric biopsy	14–80	Histology Urease test	82.2	[1]
Aseer	Histopathology-based	Patients with duodenal ulcer	126	1992–1993	Gastric biopsy	18–68	Histology Urease test	96	[18]
Aseer	Histopathology-based	Patients with GI symptoms	528	1995–1996	Gastric biopsy	≥3.0	Histology	67	[19]
Aseer	Histopathology-based	Patients with dyspepsia.	778	–	Gastric biopsy	10–100	Histology	75.4	[22]
Jazan	Histopathology-based	Patients with dyspepsia	404	2014–2016	Gastric biopsy	≥12	PCR Histology	46.5	[21]
Jazan	Urban-based	Patients with infantile colic	55 cases 30 controls	2009	Stool	2 weeks to 4 months	Stool antigen	81.8	[5]
Jazan	Histopathology-based	Patients with dyspepsia	488	1995–1998	Gastric biopsy	13–90	Histology	54.9	[20]
Najran	Histopathology-based	Obese patients	340 cases 340 controls	2013–2014	Gastric biopsy	31.54 ± 8.27 (Mean ± SD)	Histology	58	[24]
*East*									
Al-Ahasa	Histopathology-based	Obese patients	62	2006–2008	Gastric biopsy	18–51	Histology	85.5	[26]
Khobar	Histopathology-based	Chronic haemodialysis patients	54	1996–1997	Gastric biopsy	42.4 ± 18 (Mean ± SD)	Histology	63	[44]

GI: Gastrointestinal; PCR: Polimerase-chain reaction; SD: Standard deviation

#### Southern area

Numerous studies determined the prevalence of *H. pylori* infection in the Aseer and Jazan regions among patients with gastrointestinal disorders [1, 18–22]. A study was conducted in Aseer Central Hospital in the Aseer region to determine the incidence of *H. pylori* infection from endoscopic biopsies of patients (*n* ═ 528) between 1995 and 1996. Three hundred and fifty-three (67%) patients had *H. pylori* infection [19]. Similarly, another study in Abha city, the capital of the Aseer region, reported a high rate of *H. pylori* infection (82.2%) from patients with upper GIT symptoms [1]. The infection was commonly present in patients with duodenal ulceration (92.5%), duodenitis (81%), both duodenitis and gastritis (80%), gastric antral erythema (69%), and non-ulcer dyspepsia (81%) [1]. In the Jazan region, a study was carried out to determine the prevalence of *H. pylori* among patients with dyspepsia at the Gastroenterology Division, King Fahd Central Hospital, from 1995 to 1998. *H. pylori* was detected in 268 (54.9%) of gastric biopsies from 488 patients (aged 13–90 years) [20]. In a more recent study in Jazan between 2014 and 2016, the prevalence of *H. pylori* decreased to 46.5% in gastric biopsies endoscopically obtained from 404 patients with dyspepsia. The study indicated a high prevalence of *H. pylori* infection associated with gastrointestinal diseases [21]. However, chronic active gastritis was significantly found to be associated with *H. pylori* infection [21]. It is well known that gastric colonization of *H. pylori* is a relative risk factor for the development of the upper GIT. Thus, its presence should be tested during the investigation of peptic ulcer disease and other related conditions [23].

In the Najran region, a case-control study was carried out to examine the prevalence of *H. pylori* among obese (*n* ═ 340) and non-obese (*n* ═ 340) Saudi patients in the Central Hospital’s Department for endoscopy [24]. The total prevalence of *H. pylori* infection was 58% (95% confidence interval [CI] 54%–61%). The study findings indicated that obese patients presented a significantly higher prevalence rate than non-obese patients (66% vs 50%, *P* < 0.0005) [24]. A high prevalence of *H. pylori* infection among obese individuals has been documented in several studies in Saudi Arabia [25–27]. The increasing prevalence of obesity in the Saudi population and its significant association with *H. pylori* infection necessitate addressing the national public health strategy for treating and preventing obesity.

#### Central area

In central Saudi Arabia, the prevalence of *H. pylori* infection has been well documented and found to be at a high rate in Riyadh and Qassim regions [28–34]. For example, in 1990, a study of 557 individuals in Riyadh reported that the prevalence of *H. pylori* among adults was more than 70% [28]. Likewise, high incidence rates of *H. pylori* in histological specimens of patients with duodenal ulcer (73.7%), gastric ulcer, and duodenal erosions (70%) have been reported in Riyadh in 1991 [29]. A study in Riyadh showed a high prevalence of infection (67%) among a large series of patients in an urban area of Saudi Arabia [30]. The prevalence of *H. pylori* among medical students was reported as 35%, which is lower than that reported in the general Saudi population [8]. The decline in the prevalence of *H. pylori* might be due to corresponding economic improvement in Saudi Arabia over the last 30 years and the difference in laboratory techniques used to diagnose *H. pylori* infection [8].

In the Al-Qassim region, two studies determined the prevalence of *H. pylori* infection [32, 35]. Between 2007 and 2008, a study determined the seroprevalence rate of *H. pylori* among the adolescent population in Al-Qassim compared to those from Madinah and Aseer regions [32]. The study findings revealed the highest rate of *H. pylori* infection in Al-Qassim (51%) compared to Aseer (50%) and Madinah [32]. This figure is higher than the 25% *H. pylori* prevalence reported among patients with dyspepsia and epigastric pain in the Al-Qassim region using the *H. pylori* stool antigen test [35]. These differences in diagnostic tools used for examining *H. pylori* infection might reflect the variation in the prevalence rate.

#### Western area

In the western area of Saudi Arabia, there are two administrative regions, Makkah and Al-Madinah. The Makkah region has two main cities (Makkah Al-Mukaramah and Jeddah) and it is the second most populous region in the country after the Riyadh region [36]. In Makkah city, the prevalence of *H. pylori* has been documented in both symptomatic health individuals and patients with GIT disorders [4, 6, 16, 37–39] (Table 1). A study in Makkah estimated the prevalence of *H. pylori* and its relationship with chronic recurrent abdominal pain among 314 school students [37]. Overall, the *H. pylori* test was positive in 27.4% of students. There was a significant association between *H. pylori* infection and recurrent abdominal pain among school students [37]. Another study evaluated the prevalence of *H. pylori* infection and its diverse pathology among patients (*n* ═ 132) of different age groups presenting with peptic ulcer diseases. *H. pylori* was mainly detected in patients with chronic active gastritis (89%) and severe active gastritis (96%) [6]. Karima et al. [6] indicated that *H. pylori* infection was acquired early in the life of our patients, leading to multifocal pathology of the upper GIT and thus predisposing the patients to develop chronic gastritis. Another study conducted among patients at King Abdulaziz University Hospital in Jeddah found that abdominal pain and gastritis are common *H. pylori* complaints [40]. The prevalence of *H. pylori* infection has been reported among patients with iron deficiency anemia (IDA) [39]. In a cross-sectional study conducted among 79 Saudi patients with IDA from 2018 to 2020, the prevalence of *H. pylori* infection among IDA patients was high (62%). The mechanism of occult chronic GIT bleeding due to gastric mucosal destruction associated with *H. pylori* infection is a possible cause of IDA [39].

In Taif city (about 75-km southeast of Makkah), a study found that *H. pylori* was a common health problem among patients suffering from dyspepsia, with a prevalence rate of 30.1%. Active chronic gastritis was found among two-thirds (65.6%) of patients with *H. pylori* compared to 9.8% of those without *H. pylori* infection (*P* < 0.001). Therefore, screening patients with dyspepsia and active gastritis for *H. pylori* infection is recommended [41].

In Jeddah, studies have reported the prevalence of *H. pylori* infection among asymptomatic and symptomatic children [9, 42]. For example, between 2001 and 2003, a study determined 23.6% seroprevalence of *H. pylori* among asymptomatic and chronically diseased children [9]. More recently, a prospective cross-sectional study was conducted among symptomatic children (*n* ═ 303) who underwent esophagogastroduodenoscopy from 2010 to 2013. Half of the children included in this study had *H. pylori* infection [42]. Furthermore, a cross-sectional study conducted in Rabigh city (about 140-km north of Jeddah) found 51.5% *H. pylori* prevalence among intermediate schoolboys [43]. The findings of these studies might suggest a high level of *H. pylori* infection among the children population [9]. Therefore, extensive random epidemiological community-based studies including children from different age groups may provide a clear vision of *H. pylori* infection in this setting.

#### Eastern area

Three studies reported the prevalence of *H. pylori* infection among patients with comorbidities in the eastern region of Saudi Arabia and found it to be high [26, 44, 45]. Between 2006 and 2008, a retrospective study reviewed the medical records of 62 bariatric surgery patients referred for upper endoscopy in Al Ahsa province, east Saudi Arabia. The study reported a high prevalence of *H. pylori* infection (85.5%) in this group of patients [26]. Between 1996 and 1997, a prospective study determined the prevalence of *H. pylori* infection among stable chronic hemodialysis patients (*n* ═ 54) who underwent upper gastrointestinal endoscopy in Al-Khobar city, in the eastern region of Saudi Arabia [44]. *H. pylori* was detected in most patients (85.7%) with the histological diagnosis of chronic active gastritis. The prevalence of *H. pylori* infection in chronic hemodialysis patients was similar to those with normal renal function undergoing endoscopy for dyspepsia [44]. Likewise, in 2008, a study reported a similar prevalence of *H. pylori* infection among three groups of patients, those with end-stage renal disease, upper GI symptoms, and patients who had undergone kidney transplantation [45].

### Pathogenicity and role of virulence factors

*H. pylori* bacterium invades gastric mucosa or sticks to the epithelial lining of the stomach, causing both local gastric inflammation and systemic inflammation, leading to extra-GIT illnesses [7, 15]. Colonization of *H. pylori* on the gastric mucosa stimulates immune cells to release various proinflammatory substances, such as cytokines, eicosanoids, and acute phase proteins [9, 46]. Many pathologic changes occur in gastric mucosa with *H. pylori* infections, varying from active superficial gastritis to chronic active deep gastritis accompanied with intestinal metaplasia and gastric carcinoma [47]. It is well documented in the scientific literature that infection with *H. pylori* is commonly associated with chronic gastritis, peptic ulceration, cholelithiasis, gastric cancer [5, 14, 17, 27, 48–53], and extra-gastrointestinal disorders, such as unexplained IDA, idiopathic thrombocytopenia [39, 54, 55].

*H. pylori* virulence factors and their genes act as epidemiological markers and are essential to identify patients at high risk for gastroduodenal disease [11, 51]. The genetic variability of *H. pylori* has importance in determining the clinical outcome of gastric diseases [11, 56]. Several virulence factors of the *H. pylori* strain, such as vacuolating cytotoxin (*vacA*), cytotoxin-associated gene A (*cagA*), the genes induced by contact with epithelium (*iceA1* and *iceA2*), blood group antigen binding adhesion (*babA2*), and sialic acid binding adhesin involved in the pathogenesis of *H. pylori* infection [57, 58]. The *vacA* and *cagA* genes are essential in determining the clinical outcome of *H. pylori* infections and are associated with the severity of the disease [5, 10, 58]. The *vacA* gene encodes a protein-inducing vacuolation of epithelial cell cultures. There are two variable segments: the signal (s) region (s1: subtype s1a, s1b, or s2) and the middle (m) region (m1, m2) within the *vacA* gene. Specific mosaicism of these two regions of the *vacA* has been implicated in the pathogenicity of the bacterium [10]. Marie suggested that *H. pylori* strains with *vacA* type s1 and a combination of s1/m1 are associated with peptic ulceration and the presence of the *cagA* gene [11]. It has been suggested that the *cagA* gene is more prevalent in *H. pylori* isolated from patients with duodenal ulcers than in symptomatic patients with histological gastritis without ulcer [5, 11]. In addition, the *cagA* is an important marker for the most virulent strains associated with peptic ulcer, atrophic gastritis, and adenocarcinoma [11, 56].

In AL-Khobar, the eastern region, a cross-sectional study was carried out between 2020 and 2021 to determine the frequency of *cagA* and *vacA* and clarithromycin resistance of *H. pylori* isolated from gastric biopsies of patients with dyspepsia. Most isolates (97.1%, 33/34) harbored the *cagA* gene [59]. Between 2004 and 2005, a study was conducted in the western region of Saudi Arabia to determine the prevalence of *cagA+* and *iceA* genotypes among *H. pylori* isolates from a group of Saudi patients with various gastric complaints [38]. The relation between the presence of *cagA* and the development of cases of gastritis and ulcer was statistically significant (*P* ═ 0.0001). All ulcer cases were infected with the *iceA1* genotype, with a statistically significant correlation (*P* ═ 0.0001). At the same time, 94.6% of gastritis and 90.9% of normal cases were infected with the *iceA2* (*P* ═ 0.0001) genotype. In addition, combined genotypes of *cagA+/iceA1* were statistically correlated with peptic ulcer (100%) but not *cagA-/iceA1* (0%, *P* ═ 0.0001) [38]. In Jeddah, a study determined *H. pylori* virulence factors of *cagA* and *vacA* in asymptomatic *H. pylori* seropositive children. Jaber [60] found that the prevalence of *vacA* was 60%, and that of *cagA* was 56.7%, while it was 45.6% for the combined *vacA* and *cagA.*

In Riyadh, a study examined the presence of *cagA* and *vacA* genes in gastric biopsies of patients with gastroduodenal disorders [10]. A combination of *vacA* and *cagA* genes was found in 51% (60/118) of the specimens. Out of 41 patients with active chronic gastritis, 22 (54%) harbored *cagA*, and 25 (61%) had the *vacA* gene. Out of 26 (22%) patients with a duodenal ulcer, 14 (54%) had *cagA*, and 15 (58%) had *vacA* genes. Out of 18 (15%) patients with active acute gastritis, 8 (44%) were carrying the *cagA* gene, and 12 (67%) had the *vacA* gene. Moreover, the coexistence of *cagA* and *vacA* was observed in all patients with adenocarcinoma [10]. Similarly, another study in Riyadh was carried out among 68 Saudi patients to determine the correlation between *cagA* and *vacA* virulence genes and gastric clinical outcomes [11]. The *cagA* gene was detected in 42 (61.8%) of *H. pylori* isolates. The *vacA* s- and m-region genotypes were determined in all strains studied. The correlation between *cagA* and the development of cases of gastritis and ulcer was statistically significant (*P* < 0.05). In addition, significant correlations were determined between the *vacA* s1/m2 genotype and gastritis cases and between the *vacA* s1/m1 genotype and peptic ulcer cases. *H. pylori* strains of *vacA* type s1 and the combination of s1/m1 were associated with peptic ulceration and the presence of the *cagA* gene [11]. The association of toll-like receptors (TLRs) 2, 4, 9, and 10 gene polymorphisms with *H. pylori*-related gastric diseases was noted in Saudi patients. Eed et al. [51] suggested that TLR gene polymorphisms might play a role in *H. pylori* infection susceptibility and may influence its outcomes.

Gastric cancer is a significant cause of death worldwide, including Saudi Arabia. Several previous studies have reported the importance of the *H. pylori* virulence genotypes and their relation to gastric cancer and peptic ulcer diseases in the Saudi population [14, 58, 59, 61, 62]. A study was conducted at King Abdulaziz University Hospital in Jeddah between 2000 and 2014 to investigate the prevalence of *babA2*, *cagA*, *iceA1, iceA2*, *vacA* s1/s2, and *vacA* m1/m2 genotypes in *H. pylori* from gastric biopsy of patients with gastric cancer (*n* ═ 35) and gastric ulcer (*n* ═ 10) [58] The prevalence of *babA2* (100%) was significantly higher in gastric cancer than in gastric ulcer (40%) samples. The rate of virulence genes *vacA*s1 was higher in both gastric ulcer (80%) and G gastric cancer C (100%). The *vacA*sam1 and *babA2* were the most frequent genotypes and may play a role in the development of gastric cancer [58].

**Figure 3.**
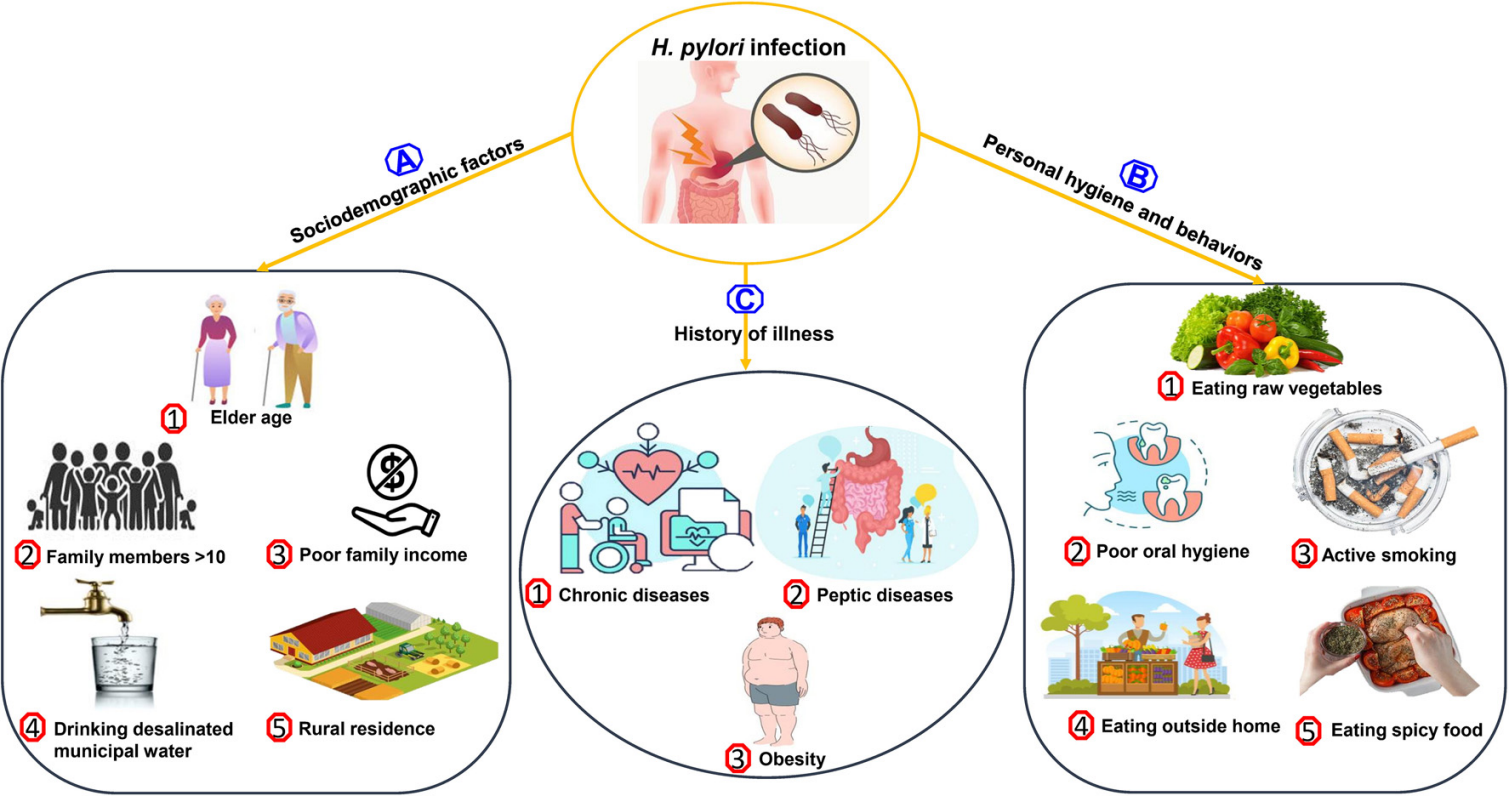
The most common risk factors associated with *H. pylori* infection in Saudi Arabia.

In Taif city in the western region, a study showed a significant association between the presence of the *cagA* gene and gastric cancer and peptic ulcer disease as well as between anti-cagA IgG and the *cagA* gene in Saudi patients. Saber et al. [62] conducted a study to assess the relationship between the occurrence of gastric cancer and peptic ulcer, the presence of *H. pylori cagA* gene and anti-cagA IgG, and to estimate their value in detecting infection by *cagA* gene-positive *H. pylori* strains in Saudi patients (*n* ═ 180). The *cagA* gene and anti-cagA IgG were found in 63.4% and 61.8% of *H. pylori*-infected patients. They were significantly (*P* < 0.01) higher in patients with gastric cancer and peptic ulcer than in those with non-ulcer dyspepsia.

In Jazan, a study identified the common *H. pylori* virulence genes among dyspeptic Saudi patients and their association with clinical outcomes and histopathological findings. Four hundred and two gastric biopsy specimens were analyzed using histopathological examination, real-time polymerase chain reaction (RT-PCR), and genotyped for *cagA, vacA*, and *iceA* genes [63]. Akeel et al. [63] found that *H. pylori* virulence genes are highly prevalent and diverse among patients with dyspepsia in southwestern Saudi Arabia. Furthermore, the *iceA* gene and the different *vacA* subtypes are significantly associated with clinical outcomes and histopathological changes, especially chronic active gastritis.

### Risk factors for *H. pylori* infection

Studies in Saudi Arabia reported various risk factors for acquiring *H. pylori* infections (Figure 3). The risk factors were mainly comprised of socioeconomic factors, history of illness, personal hygiene, and individual behaviors [9, 31, 32, 43, 64, 65]. Table 2 explores the most common predictors and risk factors associated with *H. pylori* infection from the population of different regions in Saudi Arabia. In Al-Madina, the risk of *H. pylori* seropositivity was related to socioeconomic, lifestyle, and environmental factors [64]. Significant high risk of *H. pylori* seropositivity among the healthy individuals in Al Madinah were found to be rural residence, crowded housing, low socioeconomic status, using tanks for drinking water supply, active smoking, raw vegetables, spicy food, and the presence of atopic symptoms [64]. Moreover, risk factors for acquiring *H. pylori* linked to socioeconomic status have been reported in studies from Riyadh, Al-Qassim, and Aseer [31, 32].

**Table 2:** Risk factors and predictors of *H. pylori* infection among the population in different regions of Saudi Arabia

**Area**	**Study population**	**Age range (years)**	**Risk of *Helicobacter pylori* infection**	**Predictors of *Helicobacter pylori* infection**	**References**
Riyadh	Intermediate school boys	12–15	Upper GI symptoms, recurrent abdominal pain, anorexia, nausea, family history of peptic disease, drinking desalinated municipal water, lower income, and eating outside home	Presence of any upper GI symptom, family history of peptic disease, and drinking desalinated municipal water	[43]
Jeddah	Children	≥1	Chronic diseases of diabetes, chronic asthma, chronic hemolytic anemia, neurological impairment, and Down’s syndrome	Chronic anemia and neurological impairment	[9]
Jeddah	Adults	≥40	Socioeconomic status	Male patients and those with hypertension or dyslipidemia	[27]
Aseer Al-Madinah Al-Qaseem	Student population	16–18	Socioeconomic status, gender, region	Gender (female) and region (Madinah)	[32]
Al Madinah	Healthy individuals	15–50	Age, residence, socioeconomic status, crowding index, drinking water source, sewage disposal, smoking, alcohol consumption, dietary habits, history of asthmatic symptoms	Rural residence, crowded housing, low socioeconomic status, using tanks for drinking water supply, active smoking, alcohol drinking, eating raw vegetables, eating spicy food, and presence of atopic symptoms	[64]
Najran	Obese patients	31.54 ± 8.27 (Mean ± SD)	Body mass index, gender	Body mass index	[24]
Jazan	Patients with dyspepsia	≥12	Age, histopathology findings (chronic active gastritis)		[21]
Riyadh	School-aged children	6–15	Male gender, older age, central and southern regions of Riyadh, lower socioeconomic status, and family members >10	Parents with educational level below high school, monthly family income US $ <2500, living in traditional house with limited space	[31]
Riyadh	Patients with dyspepsia	40.77 ± 14.15	Poor oral hygiene (periodontitis patients)		[68]
Makkah	Patients with epigastric discomfort	47.17 ± 9.2 (Mean ± SD )	Type 2 diabetes mellitus		[4]

GI: Gastrointestinal.

Many studies have reported on risk factors of *H. pylori* infection among school-aged children [9, 31, 43]. In Riyadh, a cross-sectional study was conducted among apparently healthy school-aged children (aged 6–15 years) for *H. pylori* seropositivity. Al-Hussaini et al. [31] identified that male gender, older age, lower levels of socioeconomic status, and >10 family members were significantly associated with *H. pylori* seropositivity. The proportion of participants with short stature was significantly higher in the *H. pylori* seropositive group than in the seronegative group (OR 1.249, 95%CI 1.020–1.531, *P* ═ 0.033). Likewise, in Rabigh, western region, the risk factors of *H. pylori* infection among intermediate schoolboys were determined. Habib et al. [43] found that *H. pylori*-infected students had significantly more association with the presence of any upper GIT symptom (*P* < 0.001), recurrent abdominal pain (*P* < 0.001), anorexia (*P* < 0.001), nausea (*P* < 0.026), family history of the peptic disease (*P* < 0.001), drinking desalinated municipal water (*P* < 0.001), lower income (*P* ═ 0.02), and eating outside the home (*P* ═ 0.003) than uninfected students. Moreover, the significant predictors of *H. pylori* infection were the presence of any GI symptom, family history of the peptic disease, and drinking desalinated municipal water [43]. Similarly, a case-control study conducted among children in Jeddah found that the risk of *H. pylori* infection was increased with chronic diseases. Moreover, the risk was significantly increased within children suffering from chronic hemolytic anemia (*P* < 0.01) and neurological impairment (*P* < 0.05) compared to controls [9].

In Makkah, a study examined the effect of age, gender, and geographical distribution on the prevalence of *H. pylori* among patients with peptic ulcer diseases. The highest prevalence of *H. pylori* was found in the younger age group, with the majority being females. *H. pylori* was mainly found in chronic active gastritis and severe active gastritis [6]. Consistently, chronic active gastritis was significantly associated with *H. pylori* infection in a study conducted in the Jazan region [21], and in the Al-Baha region, southern Saudi Arabia [66].

The association between poor oral hygiene and infection with *H. pylori* in the oral cavity has been studied [67, 68]. Studies have discovered *H. pylori* in dental plaque and saliva, linking the oral cavity as a potential reservoir or a possible route of transmission of the pathogens to other body sites [52, 67–70]. However, the oral cavity can act as an extragastric reservoir for *H. pylori* bacterium, leading to recurrent gastric infection [68, 71]. Al Asqah et al. [68] suggested that the oral cavity may be a reservoir for *H. pylori* and potentially a source of transmission or reinfection. Therefore, public awareness of proper hygiene practices must be increased to improve the situation [32].

### Diagnosis

**Figure 4.**
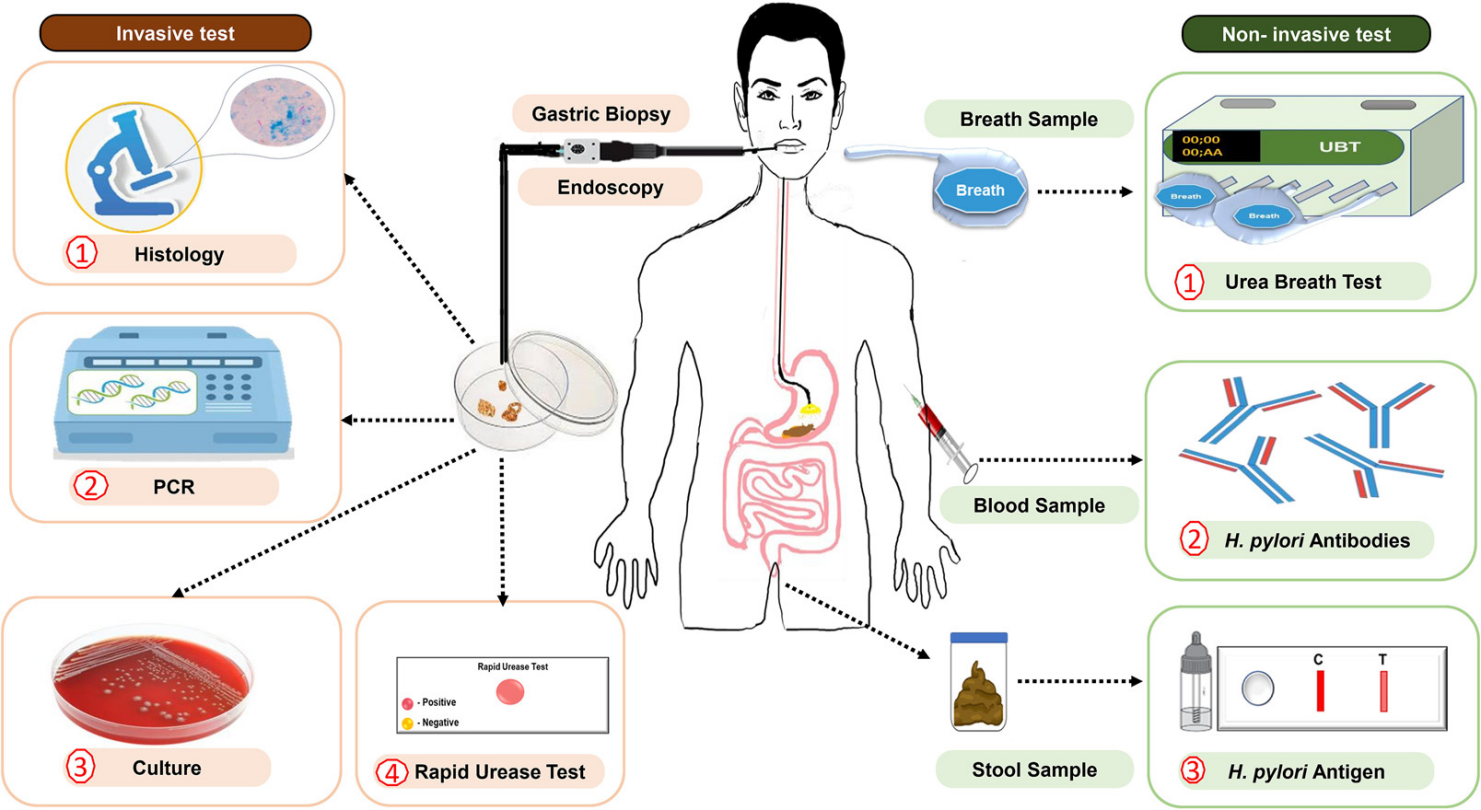
**Invasive and non-invasive assays for diagnosis of *H. pylori* infection in Saudi Arabia**.

*H. pylori* infection can be detected by various laboratory tests that have become available over recent years [67, 72, 73]. The tests can be categorized into two groups: invasive and non-invasive methods (Figure 4). Invasive techniques require endoscopy to retrieve biopsy samples for histopathological examination, rapid urease testing, bacterial culture, use of deoxyribonucleic acid (DNA) probe, brush cytology, and PCR assay for detection of 16S rRNA and urease C (*ureC*) genes of *H. pylori* [72, 74, 75]. Non-invasive methods not requiring endoscopy include testing urine, stool, blood, saliva, gastric juice, and breath samples [67, 72, 76]. The choice of appropriate test used for the diagnosis of *H. pylori* infection depends on the clinical information taken, the local availability, and the cost of individual tests [67, 72, 73, 77, 78].

#### Non-invasive and invasive tests

Non-invasive tests are simple and easier to perform in the presence of appropriate validation and standardization against known controls [42]. *H. pylori* stool antigen testing can be used in patients where invasive procedures are contraindicated and in children who do not tolerate the invasive procedures [67]. However, test-and-treat, an appropriate strategy for uninvestigated dyspepsia in young patients without alarm symptoms/signs, is recommended as the first-line strategy by all conferences and consensus [79–82].

The urea breath test is a valuable diagnostic tool for dyspeptic patients with comorbidities that increase their risk for upper endoscopy or are intolerant to upper endoscopy [76]. This test is safe, rapid, simple, and accurate in detecting *H. pylori* infection [74, 76, 83]. Serological testing is a common assay used to detect *H. pylori* antibodies from the patients’ blood sample [76]. Available serologic techniques can be applied to diagnose *H. pylori* infection, including enzyme-linked immunosorbent assay (ELISA), latex agglutination, and immunochromatography [84]. Serum antibody test results vary by geographic region and may stay positive for a prolonged period following *H. pylori* eradication, thus limiting the clinical utility for determining the presence or absence of current infection [76]. Stool antigen and urea breath tests are helpful for the defection of recent and ongoing infections [42]. Invasive assays have been considered the gold standard, but biopsy-based methods may suffer from sampling error because of the patchy nature of the infection, low concentration of bacteria in fragments, and low sensitivity culture [42].

Several studies in Saudi Arabia evaluated the accuracy of invasive and non-invasive methods in diagnosing *H. pylori* infection [42, 85–87]. A study assessed six laboratory tests to diagnose *H. pylori* infection in 303 symptomatic children who underwent esophagogastroduodenoscopy. Four invasive methods of rapid urease test, histology, antral nodularity, and biopsy culture were performed, along with two non-invasive serologic and stool antigen test methods [42]. Most of these methods showed high specificity and moderate-to-low sensitivity using positive tissue culture as a gold standard assay. Sensitivity was 62% for antral nodularity, 69% for stool antigen, and 87% for the rapid urease test. In addition, the rapid urease test showed the lowest specificity, 65%, compared to biopsy culture (88%) and histology (89%) [42]. Hasosah [42] indicated that gastric histology remains the gold standard for diagnosing *H. pylori* infection, but the rapid urease test is a valuable diagnostic method for identifying *H. pylori* with the highest sensitivity compared to antral nodularity and stool antigen test. A study found that immunohistochemical staining is an alternative diagnostic tool to PCR in detecting *H. pylori* from gastric biopsies [85]. Gastric biopsies were obtained from 50 Saudi patients with chronic gastritis and minimal or atypical infection, subjected to immunohistochemical staining, routine hematoxylin and eosin, and modified Giemsa staining. The results of staining were compared with those of quantitative RT-PCR. The quantitative RT-PCR assay identified *H. pylori* in 32 (64%) cases, whereas immunohistochemical staining, hematoxylin and eosin, and modified Giemsa staining identified *H. pylori* in 29 (58%), 27 (54%), and 21 (42%) cases, respectively. Immunohistochemical staining exhibited the highest diagnostic accuracy (90%), followed by hematoxylin and eosin (58%) and modified Giemsa staining (50%) [85].

Researchers compared the usefulness of four diagnostic methods of culture, histology, stool antigen test, and campylobacter-like organism test for *H. pylori* infection in gastric antrum mucosal biopsies from 115 Saudi patients with dyspepsia [86]. Utilizing a culture method as a gold standard assay, the sensitivity and specificity were 97.5% and 97.2% for histology, 91.9% and 98.6% for stool antigen test, and 79.7% and 97.2% for campylobacter-like organism test, respectively [86]. Al-Humayed et al. [86] found that culture, histology, and stool antigen tests all have comparable results, and there was no need to use all three at the same time for the diagnosis of *H. pylori* infection. On the contrary, another study suggested that none of the rapid urease test, histology, culture, and ELISA methods was independently sufficient to make an etiologic diagnosis of *H. pylori* infection. Moreover, Akbar and Eltahawy [87] determined the presence of *H. pylori* in 491 patients complaining of epigastric pain by three biopsy-based methods (rapid urease test, histology, and culture) and a serological test by ELISA. *H. pylori* was detected in 70% of patients examined by histology, 59% by rapid urease test, and 78% by ELISA. Osoba et al. [77] compared the value of stool antigen and campylobacter-like organism tests in diagnosing *H. pylori* infection among 60 Saudi patients with dyspepsia. The stool antigen test yielded higher sensitivity (88.6% vs 87.8%) and specificity (93.5% vs 92.5%) than the campylobacter-like organism test. The positive predictive value of the stool antigen test was 93.9% compared to 93.5% for the campylobacter-like organism test. The negative predictive value was 87.8% for the stool antigen test and 86.2% for the campylobacter-like organism test. The study findings indicated that the stool antigen test is valuable and reliable for diagnosing *H. pylori* infection.

The effectiveness of serological tests for the detection and evaluation of *H. pylori* infection has been reported by many authors [88–90]. A study compared the performance of two indirect ELISA in detecting *H. pylori* IgG antibodies in serum and saliva with endoscopic observations and histologic findings of biopsies from dyspeptic patients. Both serum and saliva samples showed high sensitivity (90.5% vs 95%, respectively) and low specificity (84.5% vs 70%, respectively) in diagnosing *H. pylori* infection. Therefore, testing *H. pylori* antibodies in serum and saliva could be an alternative to the non-invasive procedure in areas with a high incidence of infection and for children and those who resent venipuncture [88]. Another study evaluated the value of the salivary sample in detecting anti-*H. pylori* among endoscopy patients with chronic liver disease. The finding revealed low specificity (75.8%) and sensitivity (36.6%) of salivary in detecting anti-*H. pylori* antibody [67]. The usage of the rapid latex-agglutination test as a serological test for the detection of *H. pylori* infection has been evaluated in two earlier studies. One study examined the accuracy of latex-agglutination in detecting *H. pylori* among 70 patients compared to a standard biopsy-related test. The sensitivity and specificity of the latex agglutination test were 59% and 89%, respectively, indicating that this test is not recommended for the serological diagnosis of *H. pylori* infection [89]. However, a second study supported using serology as a non-invasive and rapid test for diagnosing *H. pylori* infection among dyspeptic patients in areas with low prevalence. Babay et al. [90] analyzed blood samples from 152 dyspeptic patients and 51 asymptomatic controls in a case-control study. IgG and IgA were positive in 33.5% and 41.1%, respectively, compared to 13.8% for both IgG and IgA in controls (*P* ═ 0.002). Therefore, performing blood serology testing in such situations could facilitate the application of the test-and-treat strategy [81].

### Treatment options

Antimicrobial agents are usually effective in treating *H. pylori* and associated disorders [91, 92]. Research data suggest that the success rate of classic triple therapy has decreased worldwide and in Saudi Arabia, with increased rates of resistance to clarithromycin and metronidazole [2, 91, 93, 94]. The current Maastricht V Consensus Report recommends a triple-therapy regimen containing two antibiotics (clarithromycin with amoxicillin or metronidazole) and a proton pump inhibitor as first-line treatment in regions with low clarithromycin resistance (<15%). In the area of high (>15%) or unknown clarithromycin resistance, the bismuth quadruple therapy or non-bismuth concomitant quadruple therapy is recommended for 14 days as the first-line therapy [78]. In case of bismuth-quadruple therapy failure, second-line regimens are 14-day fluoroquinolone-containing quadruple (or triple) therapy or the high-dose proton pump inhibitor–amoxicillin dual therapy are recommended [78, 95]. In cases of high fluoroquinolone resistance, combining bismuth with other antibiotics or rifabutin may be an appropriate choice [78]. However, local antimicrobial susceptibility testing and eradication rates should be carried out to evaluate the efficiency of empiric second-line therapy [78].

In Saudi Arabia, according to the recent guidelines of the Saudi Gastroenterology Association, a combination of effective antibiotics with low resistance is selected and given for 10–14 days in most regimens. Since clarithromycin resistance in Saudi Arabia exceeds 15%, quadruple therapy for 10–14 days should be considered a first- or second-line treatment [93]. A single-center randomized open-label clinical trial in Saudi Arabia found that sequential therapy for 10 days is more cost effective for the eradication of *H. pylori* compared to the 14-day sequential therapy [96]. In case of quadruple therapy failure, concomitant treatment, rifabutin-amoxicillin, or levofloxacin-based quadruple therapy for 14 days can be considered [93]. If two different regimens fail to eradicate the infection, it is recommended to perform susceptibility testing or PCR assay before administering further treatment [2, 93, 97].

Several studies on the outcome of treatment of *H. pylori* based on analysis of antibiotic resistance have been carried out in Saudi Arabia [2, 91, 93, 98–100]. Between 1998 and 2000, a study in King Abdulaziz University Hospital, Jeddah, reported that *H. pylori* resistance to metronidazole among infected patients was 80%, 4% for clarithromycin, 1.3% for amoxicillin, and 0.4% for tetracycline [91]. A prospective study was carried out among patients undergoing gastroscopy for upper gastrointestinal symptoms in Saudi Arabia to compare the efficacy of sequential vs standard triple therapy in curing *H. pylori* infections. Resistance rates have been reported for metronidazole (48.5%), clarithromycin (23.3%), amoxicillin (14.8%), levofloxacin (11.1%), and tetracycline (2.3%) [2]. Such findings indicate that clarithromycin and metronidazole resistance is common and should not be used as a single choice to treat *H. pylori* infection [2, 91].

A recent retrospective cohort study in a tertiary care hospital in Jeddah compared the eradication rate of the levofloxacin-based regimen to that of the conventional first-line clarithromycin regimen. Azab et al. [101] confirmed using a levofloxacin-based regimen as a first-line therapy in treating *H. pylori* infection for 14 days regardless of diabetes and esophagogastroduodenoscopy findings.

The resistance rate of *H. pylori* to metronidazole is increasing worldwide and in many geographical regions [98]. In Saudi Arabia, *H. pylori* resistance to metronidazole has increased over time [98, 102]. A retrospective study in the western region compared the antimicrobial susceptibility of *H. pylori* isolated between 1987 and 1988 to those subsequently isolated in 1990–1996. Metronidazole resistance was estimated at 35.2% in the first period but was elevated to 78.5% during the second period [98]. Similarly, in another study, a high resistance rate (64.4%) to metronidazole was observed among *H. pylori* isolated from patients aged between 16 and 75 years [102]. The high resistance rate of *H. pylori* to metronidazole indicates that it has become ineffective in eliminating *H. pylori*. Therefore, tetracycline can be a choice for treatment regimens in such settings [91, 98]. The resistance rates of *H. pylori* to antibiotics commonly used to treat *H. pylori* infection should be determined in each geographical region and among different population groups for proper clinical practice and management [91, 103]. However, empiric second-line and rescue therapies should be guided by local resistance patterns assessed by susceptibility testing and eradication rates to optimize treatment success [97].

## Conclusion

The prevalence of *H. pylori* infection remains high and widely distributed in Saudi Arabia. However, there is still a knowledge gap regarding *H. pylori* infection in certain geographical regions. There is considerable data in Saudi Arabia demonstrating different factors contributing to acquiring *H. pylori* infection. The study showed the diversity of *H. pylori* virulence genes with variations in geographical distribution. However, *cagA* and *vacA* are the common types associated with pathogenicity and clinical outcomes of the disease. Further studies are needed to determine the presence of virulent genes and their association with the severity of the infection and the development of gastric carcinoma. Multiple laboratory methods are used to detect *H. pylori* in Saudi Arabia. However, early infection investigation with proper techniques can reduce the burden of infection and complications. Although *H. pylori* stool antigen and urea breath tests are more accurate, simple, and safe in the detection of *H. pylori* infection, culture remains essential to determine antimicrobial susceptibility patterns of the *H. pylori* bacterium. Resistance of *H. pylori* to the traditional first choice of antibiotic treatment (clarithromycin, metronidazole, amoxicillin, and levofloxacin) has been seen at different rates in Saudi Arabia. Therefore, each local setting in the country should evaluate antibiotic resistance patterns of *H. pylori* within their local community before implementing a treatment protocol for the disease. The findings of this review highlight the importance of continuing to implement the screening, control measures, and prevention of *H. pylori* infection, in order to combat the spreading infection and other related complications.

## Data Availability

The current study was based on the results of relevant published studies.
